# Exposure to a community-wide campaign is associated with physical activity and sedentary behavior among Hispanic adults on the Texas-Mexico border

**DOI:** 10.1186/s12889-017-4893-4

**Published:** 2017-11-16

**Authors:** Natalia I. Heredia, MinJae Lee, Belinda M. Reininger

**Affiliations:** 1 The University of Texas Health Science Center at Houston, School of Public Health, Center for Health Promotion and Prevention Research, 7000 Fannin St, Suite 2576E, Houston, Texas 77030 USA; 2 Division of Clinical and Translational Sciences, Department of Internal Medicine, McGovern Medical School at The University of Texas Health Science Center at Houston, Houston, Texas USA; 3 The University of Texas Health Science Center at Houston, School of Public Health, Brownsville Regional Campus, Houston, Texas USA

**Keywords:** Community-based research, Health disparities, Intervention study, Physical activity, Sedentary behavior

## Abstract

**Background:**

Despite evidence for the use of community-wide campaigns to promote physical activity, few evaluations of community–wide campaigns in Hispanic communities exist. This study assessed the associations of exposure to a community-wide campaign with physical activity and sedentary behavior among Hispanic adults living on the Texas-Mexico border.

**Methods:**

The intervention, Tu Salud ¡Si Cuenta! (Your Health Matters!; TSSC), included a newsletter, community health worker discussion, TV and radio segments, which were conducted from 2005 to 2010. We matched an intervention (*N* = 399) and a control community (*N* = 400) on demographics and used a cross-sectional assessment in 2010 with randomly sampled adults from both communities. We collected exposure to the campaign, as well as physical activity and sedentary behavior with the International Physical Activity Questionnaire. We conducted bivariate analyses and multivariable logistic regression models to assess the association of TSSC exposure and its components with meeting moderate-to-vigorous intensity physical activity (MVPA) guidelines and exhibiting excessive sedentary behavior, controlling for covariates.

**Results:**

As compared to the control community, the intervention community has 3 times the odds of meeting MVPA guidelines (Adjusted OR [AOR] = 3.01, 95% CI = 1.85–4.88, *p* < .05) and 2 times lower odds of excessive sedentary behavior ((AOR = 0.46, 95% CI = 0.30–0.70, *p* < .05). Exposure in the intervention group to any component was associated with five times the odds of meeting MVPA guidelines (AOR = 5.10, 95% CI 2.88–9.03, *p* < .001) and 3 times lower odds of excessive sedentary behavior (AOR = 0.32, 95% CI 0.17–0.60, *p* < .001), compared with those unexposed in the control community. Exposure to newsletters, CHW discussions and TV segments were associated with significantly lower odds of excessive sedentary behavior and higher odds of meeting MVPA guidelines. Exposure to radio segments was only associated with a significantly higher odds of meeting MVPA guidelines (AOR = 4.21, 95% CI = 1.17–15.09).

**Conclusions:**

This study provides some evidence of the association of community-wide campaigns and its components in Hispanic communities with higher levels of MVPA and lower levels of excessive sedentary behavior.

**Trial registration number:**

NCT00788879 Date: November 11, 2008.

## Background

Physical activity has been associated with numerous beneficial health outcomes [1, 2]. An accumulation of international research on physical activity promotion has led to a systematic review [3] and eventual recommendations by the Guide to Community Preventive Services for increasing physical activity [4]. These recommendations include the use of community-wide campaigns [4], which are typically highly visible and employ multiple components including the use of media, social support, risk factor screening, health education and environmental changes, as a cost-effective strategy to increase physical activity [3, 5]. However, this recommendation was based on a body of work that included few studies with Hispanics, and none which were conducted recently or specifically analyzed results among the Hispanic subgroup included in the sample [6–8]. Moreover, although community-wide campaigns have been evaluated in Brazil [9, 10], to our knowledge, despite the dissemination of a successful model to other parts of Latin America [11], it has not been formally evaluated in any Spanish-speaking population. Furthermore, despite the Guide to Community Preventive Services’ review and recommendation for community-wide campaigns, other systematic reviews concluded that further research is needed to conclude that community-wide interventions are effective [12], especially with study designs that include intervention and control groups [13]. In this paper, we detail the results of a community-wide campaign designed to increase physical activity among Hispanic adults that compares intervention and control communities.

A multi-dimensional view of physical activity has recently been promoted, that is, one that recognizes that sedentary behavior is a separate behavior from physical activity [14] with independent, negative effects [15, 16]. Sedentary behavior has been independently associated with cancer risk and mortality [17–19], cardiovascular disease and mortality [20–22] and all-cause mortality [23, 24]. Currently, there are no recommendations from the Guide to Community Preventive Services for reducing sedentary behavior in adults and there is a need to test behavioral interventions and strategies to reduce sedentary behavior in adults [25, 26]. One starting point for developing behavioral strategies to reduce sedentary behavior could come from evaluating the association of existing evidence-based physical activity strategies with sedentary behavior [27], as there is some evidence that even physical activity targeted interventions can still have effects on sedentary behavior in adults [28]. In this study, we aimed to build the evidence base for the association of physical activity-targeted community-wide campaigns with lower levels of sedentary behavior.

The purpose of this paper is to compare predominantly Hispanic intervention and control communities on the Texas-Mexico border to assess the associations of being in the intervention community and exposure to a physical activity-targeted, community-wide campaign with meeting moderate-to-vigorous-intensity physical activity (MVPA) guidelines and excessive sedentary behavior. We expect that those exposed to the community-wide campaign, compared with controls or those unexposed in either condition, will participate in more MVPA and less excessive sedentary behavior. We also expect that all campaign components (newsletter, community health worker (CHW) discussion, and TV and radio segments) will be associated with both outcomes, though the more intensive, interpersonal component (CHW discussion) will produce the greatest associations with both behavioral outcomes.

## Methods

### Intervention

The Tu Salud ¡Si Cuenta! (Your Health Matters!, TSSC) community-wide campaign development and implementation from 2005 to 2010 has been described in detail elsewhere [29–31]. Briefly, the TSSC campaign was established to address low MVPA and unhealthy food choices among Mexican-American populations living on the Texas-Mexico border, who have been shown to have high obesity rates and low levels of MVPA [32, 33]. The campaign was designed and implemented by community partners, including city officials, health professionals, community leaders, and non-profit organizations. The components of the campaign included a newsletter, community health worker (CHW) discussion, and TV and radio segments. The components were delivered primarily in the Spanish language and have been layered in over the past decade, as funding and community input have allowed.

The TSSC campaign was based on the Transtheoretical Model [34] and Social Cognitive Theory [35]. The Stages of Change and the Processes of Change from the Transtheoretical Model framed campaign messages that were used in all the components of the campaign, as we focused on reaching people who were physically inactive or not active enough to meet MVPA guidelines (those in the *Precontemplation, Contemplation* and *Preparation* Stages). We relied heavily on some of the Processes of Change by creating awareness of the needs and benefits of MVPA (*Consciousness Raising*) through emotional role model stories (*Dramatic Relief*) and encouragement to envision one’s self as more physically active (S*elf-reevaluation*). Building on the cornerstone of the Social Cognitive Theory, we designed the campaign to promote *Self-efficacy,* or the confidence to be physically active in the face of obstacles. As such, the campaign messages gave specific examples of struggles commonly faced when trying to be physically active (e.g., no time, parenting/job responsibilities, no safe place) and showed how other local role models have overcome such barriers, thus aiming to improve their confidence to meet MVPA guidelines. Additional details about the behavioral change strategies incorporated into the campaign are published elsewhere [29].

### Study design

TSSC was evaluated as a quasi-experimental design with intervention (Brownsville) and control (Laredo) Texas communities. Both cities are located on the Texas-Mexico border, though at a distance of 200 miles to limit contamination. The control community was selected based on its match to the intervention community on location on Texas/Mexico border, size, percent Hispanic and low-income status. Within each city, unique panels of individuals were sampled every 2 years. These cross-sectional samples were collected at baseline (2006), first (2008) and second (2010) follow-up. At each time point the sampling frame of a neighborhood areas was matched again on size, percent Hispanic and low-income status based on the US Census data (2000) by tract and block. An adapted two-stage cluster sampling methodology was then used [36]. A random sample was drawn in each neighborhood area using a 1-in-10 systematic sampling of housing units. That is, after the closest cross-street to the center of the neighborhood area was identified, data collectors selected every 10th house in all four Cardinal Directions from that central cross-street. If an individual from that 10th house was not home, not eligible or not interested in participating, then data collectors approached the 11th house, followed by the 9th house. If none of those three houses was enrolled (to obtain the 1-in-10 house for that segment), then the data collector proceeded to the next segment of 10 homes, repeating the process.

In regards to comparability of the sample at baseline, analysis conducted on the baseline sample (2006) showed comparability of the intervention and control samples in regards to the outcome of physical activity. A single question, “During the last month, not counting your regular work, did you participate in some form of exercise such as running, calisthenics, golf, gardening or walking for exercise?” with 200 intervention and 193 control community participants responding showed no significant difference between the two communities in past month physical activity (*p* = .43), even when controlling for covariates (*p* = .26). Sedentary behavior was not assessed at baseline.

This paper examines data from the cross-sectional samples collected at the intervention and control sites at the second follow-up (2010) when exposure to TSSC in the intervention site would have accumulated. The Center for the Protection of Human Subjects at UTHealth approved this study.

### Data collection and management

Data were collected door-to-door from the randomly selected adults, 18 years and older, in either Spanish or English. Data were collected from the intervention and control communities in the same way. Households identified using the two-stage cluster sampling (detailed above) were approached up to five times at various days and times, including in the evening. Participation was limited to one adult (age 18 or older) per household, selected based on the next birthday if multiple adults were present. Written informed consent was first obtained before paper-and-pencil interviewer (PAPI) administration of the survey. When the survey was complete, the participant received a $10 gift card. Data entry was completed by trained personnel, with data checked for outliers. A 10% sample was extracted to check for accuracy; mistakes identified in that process were corrected, an additional 10% sample was checked and the process repeated until no additional mistakes were found.

### Measures

We collected information on exposure to the TSSC components, as well as on MVPA, sedentary outcomes and demographic variables.

#### Exposure

Measurement of TSSC exposure and imputation of missing values for this variable has been described elsewhere [29]. Briefly, exposure was assessed in two stages. In stage 1, participants recalled, without the use of aids, exposure to the TSSC campaign or its messages. They were then asked whether they had been exposed to each individual TSSC component, regardless of their response to the initial question. For participants in either the intervention or the control community who reported no TSSC campaign exposure, we recorded that they were not exposed to the individual TSSC components if that data were missing (*n* = 592, 74.1%).

In stage 2, participants were asked to describe one of the health messages included in TSSC. If a participant indicated he/she had been exposed to TSSC in stage 1 but was unable to confirm that exposure in stage 2, we reclassified the individual as not having been exposed (*N* = 13, 1.6%). None of the individuals who indicated in stage 1 they had not been exposed to TSSC were able to confirm exposure in the stage 2.

#### Physical activity and sedentary behavior

**Fig. 1:**
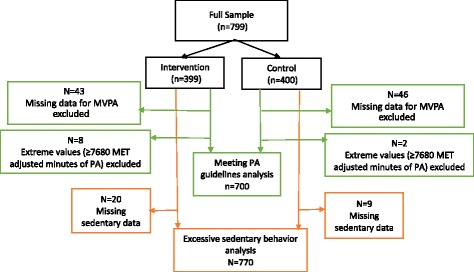
Analytic samples

At the second follow-up (2010), when additional funding was present, we assessed physical activity and sedentary behavior using the International Physical Activity Questionnaire (IPAQ) [37]. Given that our intervention was designed to impact leisure-time physical activity only, we limited our measures to that domain. For example, one question was “During the last 7 days, on how many days did you do vigorous physical activities in your leisure time?” Individuals reported the frequency and duration of MVPA in hours and minutes per week over the previous 7 days. We determined whether U.S. MVPA guidelines were met [38] by calculating the metabolic equivalent (MET) adjusted minutes of MVPA reported. Total activity < 600 MET adjusted minutes was considered as not meeting MVPA guidelines [39]. Based on the recommended scoring protocols, 10 (1.3%) participants with extreme values (≥ 7680 MET adjusted minutes) of MVPA were excluded from the analyses (Fig. 1). For a subset of the surveys (*n* = 84) the directions to data collectors indicated that if a participant reported “0” minutes of activity that a “0” should be entered into minutes of moderate activity and that no response should be entered in vigorous activity. During analysis, we recoded the missing responses for vigorous activity for this subset as “0” because this pattern represented no activity for either moderate or vigorous physical activity. Later the survey collection instructions were modified so that 0 min of moderate and vigorous activity data were actually recorded. For participants where minutes of vigorous activity were recorded (*n* = 89) but moderate activity was missing, we excluded these individuals from the analysis because there was no way to accurately estimate their level of moderate activity (Fig. 1). Previous research indicates that it is more likely for Hispanics adults to perform moderate rather than vigorous activity [40, 41], therefore making “0” minutes of moderate physical activity an unsatisfactory recoding option if it was missing when a response for vigorous activity was present.

At second-follow up (2010), participants also reported the frequency and duration of sitting in the previous 7 days, including weekdays and weekends, using the IPAQ [42]. For example, one question was “During the last 7 days, how much time did you usually spend sitting on a weekday?” with individuals reporting hours and minutes per day. Given the lack of a well-recognized cut-point for excessive sedentary behavior in the literature, we used the cut point of ≥ 540 min. This is similar to the threshold of the highest quintile for sitting time, assessed by the IPAQ in a 20-country study [43], and is similar to the cut point previously used to define excessive sedentary behavior [44]. These two studies used weekdays only to reflect usual behavior. However, given the low representation in the highest quintile for the only Spanish-speaking, low- or middle-income country included (Columbia) in the 20-country study [43], as well as the low representation above this threshold for weekday sitting in our own sample, we believe that using the combined weekday and weekend variable is appropriate and identifies extreme sedentary behavior in our sample. We excluded from the analyses 29 (3.63%) participants with missing sedentary data (Fig. 1).

#### Demographic variables

We collected demographics, which were used as covariates. Age was collected with one item, asking participants to respond how old they were. Sex was collected with one item, with the question asking “What is your sex?” Marital status was collected with one item that asked individuals to characterize themselves as married, divorced, separated, never married, living with someone, or widowed. Education was measured with one question asking “What is the highest grade or school year you completed?”. Employment was collected with one item, answered as yes or no, asking “Are you currently employed?” Lastly, given the population in the Lower Rio Grande Valley where both communities were located, ethnicity was measured with one item asking “Do you consider yourself Mexican or Mexican-American?”

### Statistical analysis

Univariable associations of demographic characteristics with the intervention community or outcome variables (MVPA and excessive sedentary behavior) were assessed using the Chi-square test for categorical variables and the Student’s *t*-test for continuous variables or their non-parametric counterparts, if necessary. Multivariable logistic regression models were conducted to assess the association of being in the intervention community with meeting MVPA guidelines and excessive sedentary behavior separately, while controlling for potential confounding factors, such as age, sex, marital status, education, employment and ethnicity.

We then analyzed the results based on actual exposure to TSSC as a whole and the individual components as we did not expect to reach all individuals within the community. Of our sample, 43.6% of the intervention community reported exposure to any component, 38.0% reported exposure to the newsletter, 20.2% to the CHW discussion, 27.4% to the TV segment and 6.8% to the radio segment. Thus, we created a collapsed variable that included the community (intervention or control) and exposure to the components, given the high collinearity between the two variables. This resulted in intervention-exposed and intervention-unexposed groups. Very few (*n* = 8) individuals in the control community reported ever being exposed to any component. Thus, our comparisons are limited to intervention-exposed versus intervention-unexposed and intervention-exposed versus control-unexposed. We also examined overlap between exposures to components; however, because exposure to two different components was never greater than 10%, and was as little 2%, we analyzed each component individually.

Multivariable logistic regression models were again used to assess the association of actual exposure to TSSC and its components with meeting MVPA guidelines and excessive sedentary behavior separately while adjusting for age, sex, marital status, education, employment, and ethnicity. Possible interaction effects were also evaluated while developing the final multivariable models. Analyses were performed using SAS 9.4 (SAS Institute Inc., Cary, NC), and all hypotheses were tested at a 5% significance level.

## Results

### Demographics

In the total sample (both groups), participants were 98% Mexican-American and preferred Spanish, with a majority being married (67%) and female (85%). Only 54% had completed grade 9 or higher, and only about 30% were employed. Despite attempts to match communities on various characteristics, the intervention and control community differed significantly by education and employment, with the control community having more educated and employed individuals (Table 1).

**Table 1:** Demographic characteristics and bivariate associations

Variable	Control	Intervention	p	Meet MVPA guidelines			Excessive sedentary		
	*N* = 400	*N* = 399							
				Yes	No	p	Yes	No	p
Age, Mean (SD)	42.7 (13.09)	44.6 (15.17)	0.122^a^	39.67 (12.27)	44.26 (14.35)	**0.002**	39.92 (12.22)	44.14 (14.25)	**0.003**
Female, n (%)	340 (85.64)	338 (84.92)	0.775^b^	88 (83.02)	508 (85.81)	0.454	107 (79.26)	549 (86.87)	**0.023**
Completed grade 9 or higher, n (%)	254 (63.66)	177 (44.47)	**<0.001** ^b^	61 (57.55)	317 (53.55)	0.447	92 (68.15)	325 (51.26)	**<0.001**
Married, n (%)	274 (69.90)	250 (63.61)	0.062^b^	65 (63.11)	398 (68.03)	0.326	88 (67.69)	419 (66.83)	0.848
Mexican origin, n (%)	388 (98.73)	376 (97.66)	0.264^b^	100 (97.09)	568 (98.27)	0.419	130 (98.48)	608 (98.22)	0.834
Spanish-language preference, n (%)	395 (98.75)	372 (97.64)	0.242^b^	104 (98.11)	568 (98.44)	0.806	131 (98.50)	612 (98.55)	0.962
Employment, n (%)	138 (34.67)	104 (26.20)	**0.009** ^b^	32 (30.19)	183 (31.02)	0.865	45 (33.33)	188 (29.70)	0.404

Note: Missing data: Age (control group *n* = 4, Intervention group *n* = 8), Sex (control group *n* = 3, Intervention group *n* = 1), Education (control group *n* = 1, Intervention group *n* = 1), Marital Status (control group *n* = 8, Intervention group *n* = 6), Ethnicity (control group *n* = 7, Intervention group *n* = 14), Language Preference (control group *n* = 0, Intervention group *n* = 18), Employment(control group *n* = 2, Intervention group *n* = 2)

Bold indicates significance; ^a^Wilcoxon rank-sum test ^b^Chi-square test

Bivariate associations of demographic variables with both MVPA and sedentary behavior indicated that age, sex and education were associated with excessive sedentary behavior, while only age was associated with meeting MVPA guidelines (Table 1). Mean values for the intervention group showed 483 min of MET-adjusted minutes of MVPA, while the control group had only 171 min of MET-adjusted minutes of MVPA during the last 7 days. Furthermore, the intervention group had a mean of 1977 min of sedentary behavior in the last 7 days, while the control group had a mean of 2785 min of sedentary behavior during the same time.

### Physical activity

At second follow-up (2010), controlling for covariates (age, sex, marital status, education, employment, and ethnicity), the intervention community had 3 times the odds of meeting MVPA guidelines as the control community, regardless of actual exposure to TSSC (AOR = 3.01, 95% CI = 1.85–4.88) (Table 2).

**Table 2:** Intent-to-Treat analyses, controlling for covariates^a^

Variable	Control	Intervention	Adjusted OR (95% CI)
	*n* = 400	*n* = 399	
Meeting MVPA guidelines, n (%)^b^	33 (9.38)	73 (20.98)	*3.01(1.85,4.88)* *
Excessive sedentary behavior, n (%)^c^	90 (23.02)	45 (11.87)	*0.46(0.30, 0.70)* *

Note: *p < .001

^a^Covariates included age, sex, marital status, education, employment, and ethnicity

^b^ *n* = 89 missing total MET data (*n* = 43 for intervention and *n* = 46 for control) and 10 were excluded due to total MET > 7680 (*n* = 8 for intervention and *n* = 2 for control); total demonimator =352 for control, 348 for intervention (see Fig 1)

^c^ *n* = 29 missing sedentary data (*n* = 20 for intervention and *n* = 9 for control); total denominator=391 for control and 379 for intervention (see Fig 1)

As compared to the intervention-unexposed, the intervention group who were exposed to any TSSC component had higher odds meeting MVPA guidelines (AOR = 2.24, 95% CI = 1.28–3.91) (Table 3). As compared to the intervention-unexposed, the intervention group who were exposed to the newsletters had higher odds meeting MVPA guidelines (AOR = 2.74, 95% CI = 1.55–4.86) and those exposed to the CHW discussion had higher odds meeting MVPA guidelines (AOR = 2.50, 95% CI = 1.31–4.77) (Table 3). Further, as compared to the control-unexposed group, the intervention community exposed to the TV segments had higher odds of meeting the MVPA guidelines (AOR = 3.52, 95% CI = 1.75–7.07) and those exposed to the radio segments had higher odds of meeting the MVPA guidelines (AOR = 4.21, 95% CI = 1.17–15.09).

**Table 3:** TSSC components associated with behavioral outcomes based on multivariable logistic regression models, adjusting for covariates^a^

Variable	Contrast: intervention-exposed vs.	Meeting MVPA guidelines^b^	Excessive sedentary behavior^c^
		Adjusted OR(95% CI)	Adjusted OR(95% CI)
Any component	intervention-unexposed	2.24 (1.28,3.91)**	0.64 (0.31, 1.30)
	control-unexposed	5.10 (2.88,9.03)***	0.32 (0.17, 0.60)***
Newsletter	intervention-unexposed	2.74 (1.55,4.86)***	0.42 (0.18, 0.95)*
	control-unexposed	6.02 (3.34,10.86)***	0.23(0.11, 0.50)***
CHW discussion	intervention-unexposed	2.50 (1.31,4.77)**	0.46 (0.16, 1.36)
	control-unexposed	6.95 (3.43,14.07)***	0.22 (0.08, 0.64)**
TV segments	intervention-unexposed	1.10 (0.58,2.09)	0.33 (0.11, 0.97)*
	control-unexposed	3.52 (1.75,7.07)***	0.17(0.06, 0.49)**
Radio	intervention-unexposed	1.36 (0.39,4.72)	0.86 (0.19, 3.99)
	control-unexposed	4.21 (1.17,15.09)*	0.37 (0.08, 1.69)

Note: *p < .05; **p < .01; ***p < .001

^a^Adjusted for age, sex, marital status, education, employment, and ethnicity

^b^Based on *n* = 700 (see Fig. 1)

^c^Based on *n* = 770 (see Fig. 1)

### Sedentary behavior

Controlling for covariates, the intervention community had about half the odds of excessive sedentary behavior than the control community, (AOR = 0.46, 95% CI = 0.30–0.70) (Table 2). Overall, TSSC was associated with lower odds of excessive sedentary behavior (Table 3). As compared to the control-unexposed group, the intervention group exposed to any component had 3 times lower odds of excessive sedentary behavior (AOR = 0.32, 95% CI = 0.17–0.60). Those in the intervention group exposed to the newsletter had lower odds of excessive sedentary behavior than the intervention-unexposed (AOR = 0.42, 95% CI = 0.18–0.95). Similarly, those in the intervention group exposed to the TV segments, as compared to the intervention-unexposed, had lower odds of excessive sedentary behavior (AOR = 0.33, 95% CI = 0.11–0.97). As compared to the control-unexposed group, the intervention group exposed to the CHW discussion had almost 5 times lower odds of excessive sedentary behavior (AOR = 0.22, 95% CI = 0.08–0.64). Exposure to radio segments in the intervention group was not associated with decreased odds when compared with the intervention-unexposed or the control-unexposed.

## Discussion

This study provides evidence of association between exposure to a community-wide campaign and meeting MVPA guidelines in Hispanic adults. It also provides additional evidence that this physical activity-targeted strategy was associated with lower levels of excessive sedentary behavior. All TSSC components were associated with higher odds of meeting MVPA guidelines and lower odds of excessive sedentary behavior, except the radio segment, when compared to either comparison group (intervention-unexposed or control-unexposed). Given that only 6.8% of the sample was exposed to the radio segments, it is not surprising that we were unable to detect sedentary behavior differences between the groups. Our hypotheses that the exposure to the CHW discussion would have the greatest association with the two outcomes was only partially confirmed. Although exposure to this component had the greatest association with meeting MVPA guidelines when comparing the intervention-exposed to the control-unexposed, this was not true for the comparison between intervention-exposed and intervention-unexposed, where newsletters had the greatest association. For excessive sedentary behavior, TV segments were the individual component with the greatest association (i.e. strongest protective effect), regardless of the comparison group.

The contrasts between the intervention-exposed and intervention-unexposed groups were not significant for several components, and when there was a significant difference, there were smaller ORs. The lack of significance and smaller ORs when comparing intervention-exposed and intervention-unexposed (as opposed to intervention-exposed to control-unexposed) could potentially be due to factors outside of the TSSC in the intervention community that influenced the outcomes but that were not captured by our data collection or controlled by our study design. For example, our method of assessing exposure to the TSSC components was conservative, requiring that an individual remember specific messaging. Perhaps TSSC messaging was unconsciously registered and acted upon by individuals in the unexposed-intervention group without being able to consciously recall TSSC components and messaging. Furthermore, there may have been positive side effects of TSSC, such as other community-led efforts or environmental changes, which led to increased activity and less sedentary behavior but that were not measured. Despite these drawbacks, our findings point to a positive association of exposure to the TSSC community-wide campaign with meeting MVPA guidelines and an inverse association of exposure to TSSC with excessive sedentary behavior.

In previous studies, mass media (radio and TV segments) [45–49], along with newsletters [50, 51], and individualized, interpersonal components akin to our CHW discussions [46, 47, 51, 52] have been successfully used in community-wide interventions and campaigns to increase physical activity in adults. However, our results differ from studies that showed no association of community-wide campaigns [53, 54] and interventions [55–58] with physical activity. Although some of these interventions that were not associated with physical activity used models or theory, such as marketing principles [56] principles of community development [57], and the Hierarchy of Effects model [59], several of these unsuccessful interventions did not use behavioral theories that are known to be useful within the context of physical activity interventions generally and even community-wide campaigns specifically, such as the Transtheoretical Model [60–62] and the Social Cognitive Theory [48, 49, 63, 64]. One unsuccessful program did use two other behavior change theories, though it is not clear how (and how well) the constructs were operationalized [53]. Another program that did use the Transtheoretical Model and was still unsuccessful [55] appears to have only used the Stages of Change, rather than also incorporating strategies elucidated by the constructs from Processes of Change, which is one reason why some physical activity interventions based on the Transtheoretical Model have inconsistent and null findings [65]. In contrast, our use of behavioral change techniques and theory [29] likely contributed to our findings [66].

There are limited community-wide campaigns with Latin Americans or Hispanics against which to compare our results. Results from Agita Sao Paulo indicated that TV and radio, among other components [11], effectively increased physical activity; however, the impact of individual components was not evaluated. One study that did include Hispanics as a sub-group successfully used TV, radio segments, newsletters and individualized discussions with participants as a whole; however, despite the increase in leisure-time MVPA overall, there was not a clear indication that adding individualized discussion to media components resulted in increased physical activity beyond the effect of the media components alone [6]. A similar result was seen in another study that included a small Hispanic sample where a support group generated little effect on physical activity above that resulting from media components. Though the additional effect of the CHW discussion over the other components was not directly assessed in our study, the significant associations of exposure to the CHW discussion and physical activity indicates that the more personal CHW discussion could complement the all-purpose print or media components. On the other hand, campaigns that have included policy and environmental changes, which often have no individualized, interpersonal discussions or support, have been successful in Latin America [67]. Further research is needed to assess the association of community-wide campaigns and their component parts with various behavioral outcomes in Hispanic and Latin American communities.

Sedentary behavior has been measured in only a few community-wide campaigns targeting physical activity in adults. For example, as in our study, De Cocker et al. showed that their physical activity–targeted campaign decreased sitting time significantly more in the intervention community as compared to the control community [68]. A recent systematic review and meta-analysis found that interventions that targeted sedentary behavior only had more consistent findings and resulted in larger reductions in sedentary time, as compared to interventions that targeted only physical activity or both physical activity and sedentary behavior [28], while yet another systematic review and meta-analysis indicated that only sedentary behavior-targeted interventions had an effect on sedentary behavior [69]. However, given the independent, detrimental effects of not meeting MVPA guidelines and exhibiting excessive sedentary behavior [70–73], even greater benefits could be achieved if we can design interventions that effectively impact both behaviors. Future community-wide campaigns could, for example, be designed to impact sedentary behavior and be tested against campaigns targeting both behaviors or physical activity only, using a stepped-wedge design or three-arm study.

### Limitations

TSSC emerged from participatory processes and community needs, and, given funding limitations, study staff were not able to conduct rigorous evaluations with extensive measures that followed the same individuals over time. Therefore, there was neither a rigorous physical activity/sedentary measure used at baseline nor could we conduct longitudinal analysis or assess change over time with stronger measures of these outcomes. Given this limitation, our use of one data collection time point limits our ability to infer causality and to say that differences between the intervention and control community were due to exposure to TSSC rather than some other factor. However, our measure of physical activity at baseline points to similarities in the two communities on the outcomes at the start of the intervention. Moreover, our exposure measure rigorously assessed exposure to TSSC, reclassifying individuals as necessary if they were unable to confirm TSSC messaging.

Furthermore, we were limited by self-report data. This is especially problematic for our two behavioral outcomes, as individuals are known to misreport MPVA and sedentary behavior [74]. Unfortunately, funds were not available to collect device-based measurements, which may provide a better indication of ambulatory movement. Conversely, devices would not be able to supply domain-specific information to assess leisure-time specific behavior [75]. A combination of the two approaches might be best for future research in this area [76].

Another limitation was our use of PAPI for data collection, as it allowed questions related to physical activity to be overlooked and left blank. Computer Assisted Personal Interviewing (CAPI) or Computer Assisted Telephone Interviewing (CATI) methods could have forced documentation on the part of the interviewer or reduced routing errors. However, given the limited resources of this project and the need to approach randomly selected individuals from specific neighborhoods within the larger the community, PAPI was the only feasible way to collect data in this study.

Despite the intention to match the intervention and control communities on important characteristics, the random samples for each community were statistically significantly different on employment and education. Lastly, despite being initially aware of the inability of community-wide campaigns to reach the full community, there were many randomly selected individuals from the intervention community that were not exposed. Fortunately, the lack of reach for the campaign resulted in the additional comparison group (intervention-unexposed) that provides a sample that was more similar on employment and education, and helps to control some threats to internal validity from history effects, or events that were naturally occurring in the region that could have affected MVPA or sedentary behavior.

## Conclusions

This study provides evidence of the association of exposure to a community-wide campaign with higher levels of meeting MVPA guidelines in Hispanics adults. We also found that exposure to the campaign was associated with lower levels of excessive sedentary behavior, potentially extending the use of these campaigns to sedentary behavior.

## Abbreviations

AOR: Adjusted Odds Ratio
CAPI: Computer Assisted Personal Interviewing
CATI: Computer Assisted Telephone Interviewing
CHW: Community Health Worker
IPAQ: International Physical Activity Questionnaire
MET: Metabolic equivalent
MVPA: Moderate-to-Vigorous Intensity Physical Activity
PAPI: Paper-and-pencil interviewing
TSSC: Tu Salud ¡Si Cuenta! (Your Health Matters!) campaign
