# Correction to: Exposure to a community-wide campaign is associated with physical activity and sedentary behavior among Hispanic adults on the Texas-Mexico border

**DOI:** 10.1186/s12889-017-4947-7

**Published:** 2017-12-01

**Authors:** Natalia I. Heredia, MinJae Lee, Belinda M. Reininger

**Affiliations:** 1 The University of Texas Health Science Center at Houston, School of Public Health, Center for Health Promotion and Prevention Research, 7000 Fannin St, Suite 2576E, Houston, TX 77030 USA; 2 Division of Clinical and Translational Sciences, Department of Internal Medicine, McGovern Medical School at The University of Texas Health Science Center at Houston, Houston, TX USA; 3 The University of Texas Health Science Center at Houston, School of Public Health, Brownsville Regional Campus, Houston, TX USA

## Correction

After publication of the article [1], it has been brought to our attention that there is an acknowledgement missing. The authors would like to add the following –

“The authors would like to thank the staff and community partners who work to implement the activities of the community wide campaign. In particular we would like to thank Lisa Mitchell-Bennett, Vanessa Saldana, Jennifer Mota, Sister Phylis Peters and the team of community health workers at UT Health and Proyecto Juan Diego who were involved in planning and gathering data.”
